# Human milk oligosaccharides, milk microbiome and infant gut microbiome modulate neonatal rotavirus infection

**DOI:** 10.1038/s41467-018-07476-4

**Published:** 2018-11-27

**Authors:** Sasirekha Ramani, Christopher J. Stewart, Daniel R. Laucirica, Nadim J. Ajami, Bianca Robertson, Chloe A. Autran, Dhairyasheel Shinge, Sandya Rani, Sasirekha Anandan, Liya Hu, Josephine C. Ferreon, Kurien A. Kuruvilla, Joseph F. Petrosino, B. V. Venkataram Prasad, Lars Bode, Gagandeep Kang, Mary K. Estes

**Affiliations:** 1 Department of Molecular Virology and Microbiology, Baylor College of Medicine, Houston, 77030 TX USA; 2 Alkek Center for Metagenomics and Microbiome Research, Department of Molecular Virology and Microbiology, Baylor College of Medicine, Houston, 77030 TX USA; 3 Institute of Cellular Medicine, Newcastle University, Newcastle upon Tyne, NE2 4HH UK; 4 Department of Pediatrics and Larsson-Rosenquist Foundation Mother-Milk-Infant Center of Research Excellence, University of California, San Diego, La Jolla, 92093 CA USA; 5 Division of Gastrointestinal Sciences, Christian Medical College, Vellore, 632004 India; 6 Verna and Marrs McLean Department of Biochemistry and Molecular Biology, Baylor College of Medicine, Houston, 77030 TX USA; 7 Department of Pharmacology and Chemical Biology, Baylor College of Medicine, Houston, 77030 TX USA; 8 Department of Neonatology, Christian Medical College, Vellore, 632004 India; 9 Translational Health Science and Technology Institute, Faridabad, 121001 India; 10 Department of Medicine – Gastroenterology and Hepatology, Baylor College of Medicine, Houston, 77030 TX USA

## Abstract

Neonatal rotavirus infections are predominantly asymptomatic. While an association with gastrointestinal symptoms has been described in some settings, factors influencing differences in clinical presentation are not well understood. Using multidisciplinary approaches, we show that a complex interplay between human milk oligosaccharides (HMOs), milk microbiome, and infant gut microbiome impacts neonatal rotavirus infections. Validating in vitro studies where HMOs are not decoy receptors for neonatal strain G10P[11], population studies show significantly higher levels of Lacto-N-tetraose (LNT), 2’-fucosyllactose (2’FL), and 6’-siallylactose (6’SL) in milk from mothers of rotavirus-positive neonates with gastrointestinal symptoms. Further, these HMOs correlate with abundance of *Enterobacter*/*Klebsiella* in maternal milk and infant stool. Specific HMOs also improve the infectivity of a neonatal strain-derived rotavirus vaccine. This study provides molecular and translational insight into host factors influencing neonatal rotavirus infections and identifies maternal components that could promote the performance of live, attenuated rotavirus vaccines.

## Abstract (web summary)

Neonatal rotavirus infections are associated with gastrointestinal symptoms in some settings, but the role of host factors in clinical presentation is unclear. Here, Ramani et al. show that human milk oligosaccharides and microbiome are associated with symptomatic infection with neonatal strain G10P[11].

## Introduction

Breast milk is an excellent source of nutrition for a newborn infant, providing macronutrients such as lipids, fats, proteins, and carbohydrates, as well as numerous micronutrients essential for infant growth. In addition, breast milk contains several biologically active components such as immunoglobulins, growth hormones, oligosaccharides, and microbiota that play critical roles in infant intestinal homeostasis and immune development^1^. Among the bioactive components, human milk oligosaccharides (HMOs) are the third most abundant solid component after lactose and lipids. These unconjugated complex glycans act as prebiotics, antiadhesives, and antimicrobials and play critical roles in altering epithelial and immune cell responses^2^. HMOs are composed of five monosaccharide building blocks, including glucose (Glc), galactose (Gal), *N*-acetylglucosamine (GlcNAc), fucose (Fuc), and sialic acid (Sia). Over 100 HMOs have been structurally characterized; all contain lactose at the reducing end and are extended into complex structures by the addition of type 1 (Galβ1-3GlcNAc) or type 2 (Galβ1-4GlcNAc) chains, fucosylation, or sialylation^3^. The total amount and composition of HMOs varies between women and are dependent on maternal genetics, environment, and geographic location^4^.

HMOs act as soluble decoy receptors and block the binding of enteric pathogens such as rotavirus and *Campylobacter jejuni* to structurally analogous glycan receptors on epithelial cells^5,6^. HMOs also have indirect effects on the gastrointestinal epithelium, such as modulation of intestinal cell differentiation and apoptosis, which in turn can affect susceptibility to infectious agents^7^. Importantly, HMOs are natural prebiotics and act as metabolic substrates for specific commensal bacteria^8^; some bacteria like *Bifidobacterium infantis* directly metabolize complex HMOs^9^, while other microbial communities act in a concerted manner to sequentially degrade and metabolize complex HMO structures^10^. Thus HMOs, possibly together with the microbiota in breast milk^11,12^, play important roles in shaping the infant gut microbiome, modulating enteric infections, and protecting the newborn.

Among enteric pathogens, rotaviruses are a leading cause of severe dehydrating gastroenteritis in children under the age of 5 years worldwide^13^. Unlike infections in older children where multiple strains cause diarrhea and vomiting, neonatal infections are predominantly asymptomatic and are often associated with unusual strains that are geographically restricted^14^. In some settings, neonatal rotavirus infections have also been associated with severe gastrointestinal diseases, including diarrhea, feed intolerance, and necrotizing enterocolitis^15,16^. However, little is known about host factors mediating differences in clinical presentations. In this study, we focused on a unique rotavirus strain called G10P[11] that has been associated with a stable and high incidence of almost exclusive neonatal infections in Vellore, India^15^. In this setting, G10P[11] is associated with both severe gastrointestinal symptoms as well as asymptomatic infections^15^. We previously determined that the neonatal predilection of G10P[11] is due to the binding of the VP8* domain of the outer capsid protein VP4 to developmentally regulated precursor histo-blood group antigens (HBGAs) present on the gastrointestinal epithelium^17^. However, why G10P[11] was associated with symptomatic infections in some neonates while others were asymptomatic remained unclear.

Analysis of virological factors including whole-genome characterization of virus from asymptomatic and symptomatic neonates, examination of differences in viral load and virus shedding, and the role of the environment and care givers in virus transmission did not provide insight into differences in clinical presentations^18–20^. Since structures analogous to precursor HBGAs are present in human milk and P[11] VP8* binds HMOs on a shotgun milk glycan array^21^, we hypothesized that HMOs act as decoy receptors, competitively inhibiting the binding of G10P[11] to intestinal HBGAs and that differences in expression of such HMOs may explain the differences in clinical presentation between neonates. Multidisciplinary studies including in vitro infectivity assays, nuclear magnetic resonance (NMR), and analysis of samples from a cohort of mother–infant pairs demonstrate that, contrary to our hypothesis, HMOs are not decoy receptors for G10P[11] and provide an unexpected new insight into the pathogenesis of neonatal enteric infections. Of public health importance, HMOs enhance the infectivity of a licensed P[11] rotavirus vaccine, highlighting maternal factors that could promote the performance of live, attenuated vaccines.

## Results

### HMOs enhance neonatal G10P[11] infectivity in vitro

The VP8* domain of the spike protein of G10P[11] virus binds both type I and type II HMOs on a shotgun milk glycan array^21^. X-ray crystallographic studies of P[11] VP8* in complex with type I and type II HMOs [Lacto-*N*-tetraose (LNT) and Lacto-*N*-neotetraose (LNnT), respectively] revealed a previously uncharacterized receptor-binding site in rotavirus VP8*^22^. To elucidate the biological relevance of these interactions and determine whether HMOs act as soluble decoy receptors for G10P[11], infectivity assays were carried out on African green monkey kidney epithelial cells (MA104 cells), a well-established model for rotavirus studies in vitro, using increasing concentrations of LNT and LNnT. The highest concentrations tested were reflective of average biological concentrations in breast milk from healthy mothers^4^.

**Fig. 1:**
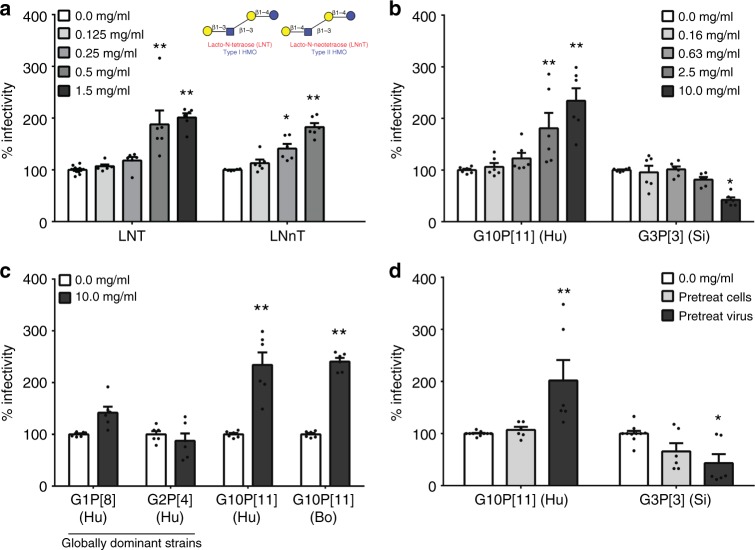
Human milk oligosaccharides are not decoy receptors for G10P[11] rotavirus. A dose-dependent enhancement in G10P[11] infectivity was seen with **a** type I HMO Lacto-*N*-tetraose (LNT) and type II HMO Lacto-*N*-neo-tetraose (LNnT) as well as **b** pooled HMOs. The structures of LNT and LNnT are shown in **a**, with the blue circle representing glucose, yellow circle representing galactose, and the blue square representing *N*-acetylglucosamine. This effect was specific to P[11] rotaviruses when compared to the reduction in infectivity for a simian (Si) G3P[3] rotavirus (**b**) and other globally dominant human rotavirus strains G1P[8] and G2P[4] (**c**). The HMO effects are mediated by an effect on the virus rather than the cells (**d**). All bars represent the mean percentage of infectivity with error bars denoting standard error of mean from a minimum of three independent experiments. No HMO treatment was considered 100% infectivity. The mean baseline titers [expressed in fluorescent focus units (FFU) per ml] for each virus strain used to denote 100% infectivity in this figure are: human G10P[11]—8.0 × 10^5^ FFU/ml; bovine G10P[11]—8.2 × 10^5^ FFU/ml; G3P[3]—2.4 × 10^7^ FFU/ml; G1P[8]—1.2 × 10^7^ FFU/ml; G2P[4]—6.0 × 10^6^ FFU/ml; *p* value <0.05 (analysis of variance with Dunnett’s post hoc test) was considered statistically significant (*p < 0.05, **p < 0.001)

Contrary to our hypothesis, a dose-dependent enhancement in G10P[11] infectivity was seen with LNT and LNnT (Fig. 1a), with the greatest increase at biologically relevant concentrations. These data suggested that LNT and LNnT are not decoy receptors for G10P[11]. However, human breast milk contains over 100 different oligosaccharides, including other structures similar to developmentally regulated precursor HBGAs with potential decoy activity. We next evaluated whether a combination of HMOs including the presence of more complex sugars leads to decoy receptor activity by testing the effect of pooled HMOs obtained from donor milk samples. The highest concentration of pooled HMOs used was representative of the average total HMO concentration in mature milk^23^. A dose-dependent enhancement in G10P[11] infectivity was seen with pooled HMOs in MA104 cells (Fig. 1b), similar to the results with individual HMOs. By contrast, a dose-dependent reduction in infectivity was observed with a commonly used laboratory G3P[3] rotavirus of simian origin (strain SA11-4F), tested as a control, suggesting that the enhanced infectivity could be specific to the neonatal P[11] strain.

Infectivity assays were therefore carried out using two globally dominant human rotavirus strains G1P[8] (strain Wa) and G2P[4] (strain DS1), as well as a G10P[11] rotavirus strain of bovine origin (strain B223). G10P[11] strains are a common cause of diarrhea in cattle^24–26^, and phylogenetic analyses show that human neonatal G10P[11] strains are most closely related to G10P[11] strains that cause diarrhea in cattle^19^. Pooled HMOs had no significant effect on the infectivity of the globally dominant P[4] and P[8] human rotavirus strains while the infectivity of both the human neonatal and bovine P[11] viruses was significantly enhanced (Fig. 1c). These data confirm that HMO-mediated enhancement in virus infectivity is specific to P[11] rotaviruses. Since all HMOs contain lactose at the reducing end, infectivity assays were carried out using lactose at the highest concentration in pooled HMOs. The addition of lactose alone did not recapitulate the enhanced infectivity seen with LNT, LNnT, or the pooled HMOs (Supplementary Figure 1). To gain preliminary mechanistic insight into the mode of action, either viruses or cells were preincubated with pooled HMOs prior to infectivity assays. Pretreated cells were washed before inoculation with virus to ensure the absence of HMO during infection. In both experimental conditions, no HMOs were included during the course of infection. Irrespective of whether there was enhancement in infectivity as seen with G10P[11] or reduction in infectivity as seen with SA11-4F, the outcome was mediated by the effect of HMOs acting directly on the virus rather than on the cells (Fig. 1d).

### Specific HMOs correlate with symptomatic infections

There are no animal models to study the human neonatal G10P[11] rotavirus. Therefore, to directly determine the biological and clinical significance of the in vitro findings for neonates, a cohort of 181 mother–infant pairs was recruited from the neonatal nurseries of the Christian Medical College (CMC) in Vellore, India, where a high incidence of neonatal G10P[11] infections has been described over a long period of time^15^. Based on clinical presentations and detection of rotavirus in stool samples from the neonates, the samples were classified into 3 groups: symptomatic rotavirus positive (*n* = 56), asymptomatic rotavirus positive (*n* = 60), and rotavirus negative (*n* = 65). HMO profiles of breast milk samples were characterized for 167 samples using high-performance liquid chromatography (HPLC)^27^.

**Fig. 2:**
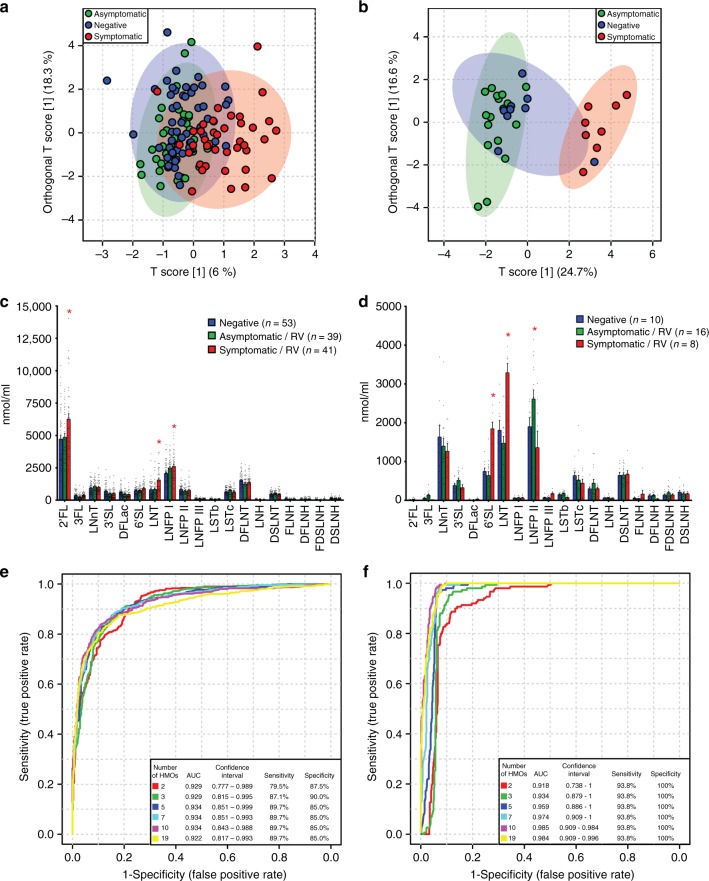
Specific HMOs are associated with symptomatic rotavirus infection. Orthogonal partial least squares-discriminatory analysis (OPLS-DA) within **a** secretors only and **b** non-secretors only show that the HMO profile of breast milk from mothers in the symptomatic rotavirus group are distinct from the asymptomatic and rotavirus negative groups. This distinction is mediated by differences in the levels of LNT, 2’FL, and LNFP I in secretors (**c**) and by LNT, 6’SL, and LNFP II in non-secretors (**d**). Bar graphs represent average levels of each HMO in nmol/ml with error bars indicating standard error of the mean. *p* value <0.05 (analysis of variance with Dunnett’s post hoc test) was considered statistically significant (asterisk (*)). The HMO profile of breast milk was predictive of symptomatic rotavirus infections as seen by receiver operating characteristic (ROC) curves generated by linear support vector machine (SVM) classification showing the area under the curve (AUC) with different numbers of HMOs included in the model within secretors (**e**) and non-secretors (**f**)

The HMO profile is influenced by maternal genetics; specifically, the presence of α(1,2) fucosylated HMO depends on whether the mother is genetically a secretor (expresses a functional α(1,2) fucosyltransferase, FUT2 enzyme) or is a non-secretor and does not express α(1,2) fucosylated HBGAs on epithelial surfaces and in mucosal secretions^28^. As anticipated, breast milk from secretor mothers contained significantly higher levels of α(1,2) fucosylated HMOs such as 2’-fucosyllactose (2’FL) and lacto-*N*-fucopentaose I (LNFP I) that were nearly absent in milk from non-secretors (Supplementary Figure 2). Regardless of maternal secretor status, the HMO profile of breast milk was similar between mothers of rotavirus-negative and asymptomatic rotavirus-positive neonates, and significantly different from mothers of symptomatic rotavirus-positive neonates (Fig. 2a, b, 2000 permutations, *p* < 0.001). Since single amino acid substitutions in the FUT2 gene lead to very different overall HMO compositions, the HMO profiles of secretors and non-secretors were independently analyzed to determine whether specific HMO are associated with rotavirus disease. Among secretor mothers, the levels of 2’FL and LNT were significantly higher in the symptomatic rotavirus group while the level of LNFP I was higher among rotavirus positives compared to the rotavirus negative group (Fig. 2c). Among non-secretor mothers, the levels of 6’-sialyllactose (6’SL) and LNT were significantly higher in the symptomatic rotavirus group while the level of LNFP II was significantly higher in the asymptomatic rotavirus-positive group (Fig. 2d). There were no overall differences in rates of symptomatic infections based on maternal secretor status since LNT is present in milk from all mothers.

To determine how predictive individual HMOs are for symptomatic rotavirus infection, receiver operating characteristic (ROC) curves were generated by linear support vector machine (SVM) classification. Since the rotavirus-negative and asymptomatic rotavirus-positive groups were highly comparable overall, these groups were combined to create a binary grouping for SVM analysis. ROCs curves plotted for the top 2, 3, 5, 7, 10, and 19 HMOs showed that the area under the curve (AUC) with only 2 HMOs was 0.929 for secretors and 0.918 for non-secretors (Fig. 2e, f, respectively). The model showing the highest accuracy for secretors was achieved at 3 HMOs (sensitivity 87%, specificity 90%, AUC 0.934; Fig. 2e). For non-secretors, the sensitivity of 93.8% and specificity of 100% with 2 HMOs did not improve in subsequent models containing up to 19 HMOs (Fig. 2f). These data suggest that evaluation of a limited number of HMOs was sufficient to predict symptomatic rotavirus infection in neonates. LNT was the most discriminatory HMO in both secretors and non-secretors by linear SVM, with this HMO being selected as one of the most important in all (100%) of the models (Supplementary Figures 3A and 3B, respectively).

**Fig. 3:**
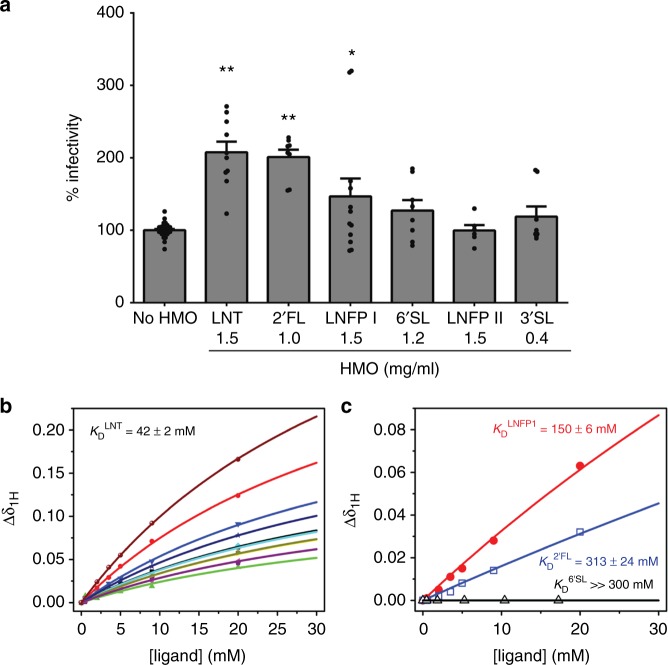
Variable effects of HMOs identified from field studies on rotavirus infectivity and VP8* binding. Of the HMOs identified as associated with rotavirus infections in the field studies, only 2’FL and LNFP I but not 6’SL and LNFP II enhance G10P[11] infectivity in vitro (**a**). LNT was included as a positive control while 3’SL that was not associated with rotavirus infections in the field studies was included as a negative control. All bars represent the mean percentage of infectivity with error bars denoting standard error of the mean from a minimum of three independent experiments. No HMO treatment was considered 100% infectivity. The mean baseline titer of G10P[11] used in these experiments (100% infectivity) was 8.8 × 10^5^ fluorescent focus units (FFU) per ml. *p* value <0.05 (analysis of variance with Dunnett’s post hoc test) was considered statistically significant (*p < 0.05, **p < 0.001). Nuclear magnetic resonance (NMR) analysis shows differences in binding of HMOs to P[11] VP8* (**b**, **c**). The highest binding was seen with LNT (**b**) while no binding was seen with 6’SL (**c**). The binding curves of NMR titrations of LNT to P[11] VP8* for nine representative NMR peaks of changes in proton chemical shifts are shown in different colors (**b**). The binding curves of NMR titrations of LNFP1, 2’FL, and 6’SL to P[11] VP8* for the same NMR peaks (greatest chemical shift change) are shown in red, blue, and black, respectively (**c**)

To determine the functional role of 2’FL, LNFP I and II, and 6’SL identified from the population studies, the average biological concentration of these HMOs was tested in infectivity assays with the neonatal G10P[11] virus. Significant enhancement in infectivity was seen with 2’FL and LNFP I but not with 6’SL and LNFP II (Fig. 3a). To determine whether enhanced infectivity is a function of VP8*–HMO binding, the binding constant (*K*_d_) was determined using NMR spectroscopy by monitoring the chemical shift changes as a function of increasing ligand concentrations for LNT, 2’FL, LNFP I, and 6’SL. The data were globally fitted to calculate a *K*_d_ of 42 ± 2 mM for LNT (Fig. 3b), 150 ± 6 mM for LNFP I, and 313 ± 24 mM for 2’FL (Fig. 3c). The highest binding was seen with LNT, which was the most discriminatory HMO in both secretors and non-secretors while no binding was seen with 6’SL (Fig. 3c). The representative NMR peaks displayed significant chemical shift changes (^1^H dimension) in the two-dimensional (2D) ^1^H-^15^N heteronuclear single quantum coherence (HSQC) spectra in the presence of the HMOs (Supplementary Figure 4A-D), revealing the direct molecular interactions between ^15^N-labeled P[11] VP8* and LNT, 2’FL, and LNFP I in solution.

### *Enterobacter/Klebsiella* correlate with symptomatic infection

Since HMOs can potentially affect pathogen infectivity by indirect mechanisms such as modulation of the infant gut microbiome, we next characterized the breast milk and infant stool microbiome from mother–infant samples. Unlike HMOs, there were no differences in breast milk or infant fecal microbiome based on maternal secretor status (Supplementary Figure 5A–B and 5C–D, respectively). The absence of difference in infant microbiota based on maternal secretor status is different from studies in other populations; however, maternal secretor status is one of many factors influencing the infant gut microbiome, and it is likely that other population-specific factors may play a role in this difference. Indeed, a recent study in 1190 healthy adults showed that FUT2 genotype and secretor status may not be associated with fecal microbial composition^29^.

**Fig. 4:**
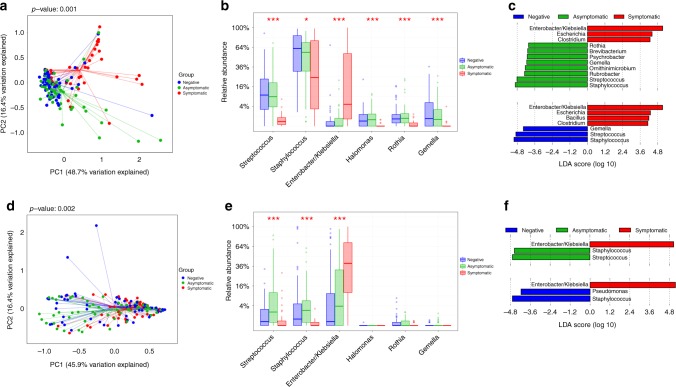
Breast milk and infant stool microbiome are associated with symptomatic infection. Weighted UniFrac principal coordinate analysis (PCoA) of breast milk microbiome show significant differences between milk from mothers of symptomatic neonates when compared to mothers of asymptomatic and rotavirus negative neonates (**a**). Bar plots show the relative abundance and significance of the most significant bacteria genera in breast milk. The center line denotes the median, the boxes cover the 25th and 75th percentiles, and the whiskers extend to the most extreme data point, which is no more than 1.5 times the length of the box away from the box. Points outside the whiskers represent outlier samples (**b**). Linear discriminant analysis (LDA) effect size (LEfSe) of bacterial genera corroborate these findings (**c**). Similar data are shown for infant fecal microbiome with PCoA in **d**, relative abundance in **e**, and LEfSe analysis in **f**. For relative abundance studies, *p < 0.05 and ***p < 0.001 are statistically significant based on Kruskal–Wallis test with FDR. For LEfSe analysis, genera with an LDA cutoff of 4.0 and significance threshold of 0.05 are shown

In accordance with orthogonal partial least squares-discriminatory analysis (OPLS-DA) of the HMO dataset (Supplementary Figure 2), weighted UniFrac principal coordinate analysis (PCoA) ordination showed that breast milk from mothers of symptomatic neonates clustered distinctly from the asymptomatic and rotavirus-negative groups (permutational multivariate analysis of variance (Permanova), *p* = 0.001). Similar to the HMO results, the latter two groups were more similar to one another and showed comparable microbiome profiles (Fig. 4a). Relative abundance analysis of discriminatory genera showed that, of the three most abundant genera, the relative abundance of *Enterobacter*/*Klebsiella* (indistinguishable based on 16S V4 region used for sequencing) was significantly higher in breast milk of mothers of symptomatic neonates [Kruskal–Wallis (KW) test with false discovery rate (FDR), *p* < 0.001], while *Streptococcus* and *Staphylococcus* spp. were significantly lower (KW test with FDR, *p* < 0.001 and 0.03, respectively) (Fig. 4b). Linear discriminant analysis (LDA) effect size (LEfSe)^30^ of bacterial genera analysis corroborate these findings with *Enterobacter*/*Klebsiella* identified as the most discriminatory genera in symptomatic group and *Staphylococcus* and *Streptococcus* identified as the most discriminatory genera in the rotavirus-negative and asymptomatic groups (Fig. 4c). To determine how predictive the 16S rRNA data are for symptomatic rotavirus infection, ROC curves were generated by linear SVM classification (Supplementary Figure 6). In concordance with the LEfSe analysis, *Enterobacter/Klebsiella* was the main genera driving the prediction of symptomatic infections.

Analysis of the infant fecal microbiome showed that the symptomatic rotavirus-positive neonates were significantly different (*p* = 0.002) compared to rotavirus-negative and asymptomatic neonates (Fig. 4d). Similar to the breast milk microbiome, the relative abundance of *Enterobacter*/*Klebsiella* was significantly higher in symptomatic neonates (KW test with FDR, *p* < 0.001; Fig. 4e). LEfSe analysis again corroborated these findings, with *Enterobacter*/*Klebsiella* identified as the most discriminatory genera in symptomatic neonates and *Staphylococcus* as the most discriminatory genera in rotavirus-negative and asymptomatic infants (Fig. 4f). These data indicate a strong association of *Enterobacter*/*Klebsiella*, whether in breast milk or in infant stool, with gastrointestinal disease presentations in neonates and a potential protective effect of *Staphylococcus* and *Streptococcus* against rotavirus infection and disease.

### HMO–microbiome correlations seen in symptomatic infection

**Fig. 5:**
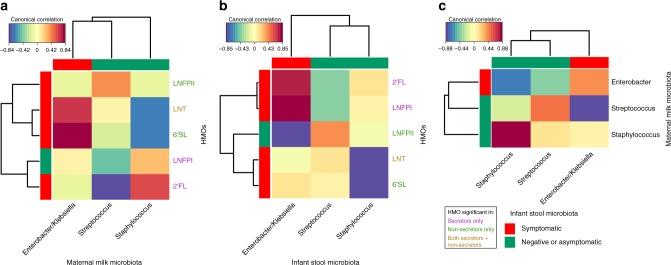
Correlations between HMOs and microbiome influence symptomatic infections. Canonical correlations between HMOs and maternal breast milk microbiome (**a**), between HMOs and infant stool microbiome (**b**), and between HMOs, maternal breast milk microbiome, and infant stool microbiome (**c**). The HMO data are based on nmol/ml concentrations in breast milk while microbiome data is based on genus relative abundance. Bars surrounding the plots at the base of the dendrogram are colored according to significant associations with symptomatic (red) or negative/asymptomatic (green) infants. HMO name color indicates if the HMO was significantly associated with secretors only (purple), non-secretors (green), or both (yellow)

Population studies individually showed the association of HMOs and the microbiota with symptomatic rotavirus infections. To evaluate the interplay between these factors, we performed sparse partial least squared (sPLS) canonical analyses to determine correlations between concentrations of the five significant HMOs identified in the field studies (described in Fig. 2) and the relative abundance of the three principally significant and discriminatory genera identified from the microbiome analyses (described in Fig. 4). Overall, the HMOs significantly associated with symptomatic infections in the neonates were positively correlated with *Enterobacter*/*Klebsiella* and/or negatively correlated with *Staphylococcus* and *Streptococcus* in breast milk (Fig. 5a) and infant stool (Fig. 5b). For example, LNT and 6’SL that are associated with symptomatic infections were both positively correlated with *Enterobacter*/*Klebsiella* and negatively correlated with *Staphylococcus* in breast milk (Fig. 5a). While LNT was directly found to enhance virus infectivity in vitro, these analyses indicate potential roles for microbiome modulation by HMOs such as 6’SL that were associated with symptomatic infections but without a direct effect on G10P[11] infectivity. Similarly examining correlations between infant stool microbiome and HMOs (Fig. 5b), the negative correlations between LNT and 6’SL in breast milk and *Staphylococcus* continued to be seen for the fecal microbiome while the positive correlations with *Enterobacter*/*Klebsiella* were lost. Conversely, 2’FL and LNFP I were positively correlated with *Enterobacter*/*Klebsiella* in stool samples. LNFP II was the only HMO significantly associated with asymptomatic rotavirus infections and showed weak correlations with both breast milk and infant fecal microbiomes. Finally, as would be expected if the breast milk microbiome contributed to colonization of the infant gut, comparing the relative abundance of the bacterial genera between breast milk and infant stool showed that each genus was positively correlated with itself between the sample types (Fig. 5c).

### HMOs enhance infectivity of a live, oral rotavirus vaccine

**Fig. 6:**
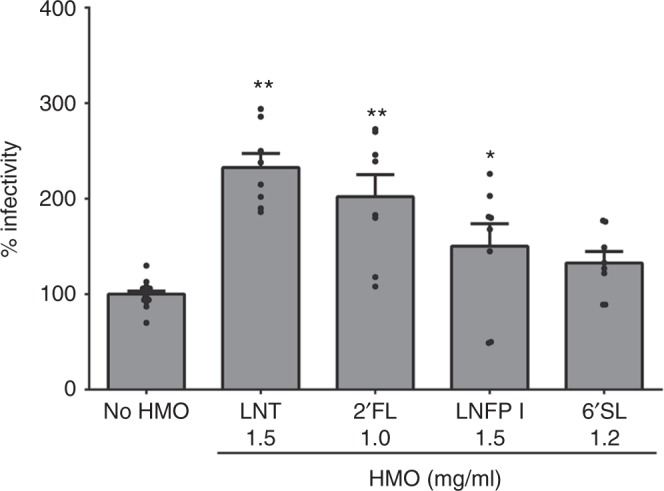
Specific HMOs enhance the infectivity of a P[11] rotavirus vaccine. HMOs 2’FL and LNT enhance the infectivity of Rotavac® in MA104 cells. All bars represent the mean percentage of infectivity with error bars denoting standard error of the mean from a minimum of three independent experiments. No HMO treatment was considered 100% infectivity. The mean baseline titer of Rotavac® (100% infectivity) was 7.3 × 10^5^ fluorescent focus units (FFU) per ml. *p* value <0.05 (analysis of variance with Dunnett’s post hoc test) was considered statistically significant (*p < 0.05, **p < 0.001)

All rotavirus vaccines currently used worldwide are live, attenuated viruses recommended to be administered at 6, 10 and 14 weeks of age^31^ where breast milk components are likely to play a critical role. A naturally attenuated neonatal virus strain belonging to the P[11] VP4 genotype (G9P[11], strain 116E) is being used as a rotavirus vaccine in India (Rotavac®) and has recently received World Health Organization (WHO) prequalification for global use^32,33^. Since Rotavac® and the G10P[11] strain described in this study share the same P[11] VP4 type^19^, we tested the effect of HMOs identified from our field studies on the infectivity of Rotavac® in MA104 cells. Similar to the neonatal G10P[11] virus, an enhancement in Rotavac® infectivity was seen with biological concentrations of LNT, 2’FL, and LNFP I but not with 6’SL (Fig. 6). These data suggest that maternal HMO profiles, both through direct effects on vaccine replication and/or possibly through indirect effects mediated by the microbiome, may be an important and previously unexplored factor contributing to differences in rotavirus vaccine take between children.

## Discussion

Breast milk is dynamic and has evolved over millennia to provide nourishment for the newborn infant. The antimicrobial and immunomodulatory effects of bioactive components such as HMOs are key for protection of the infant from infectious agents at a developmental stage where the infant’s own immune system is relatively immature and naive. However, susceptibility to infection and clinical outcome is also modulated by how the pathogen counters host restriction. This study exemplifies microbial success in an evolutionary arms race between host and pathogens. While the pathogen appears to exploit abundantly available breast milk factors to cause disease in neonates, this dominance is limited to a highly age-restricted neonatal niche, perhaps suggesting an evolutionary cost for this adaptation.

A key strength of this study is the validation of in vitro findings in a relevant human population, thereby addressing long-standing clinical and epidemiological questions on neonatal rotavirus infections and disease. For decades, it has been evident that neonatal rotavirus infections are clinically and epidemiologically distinct from infections in older children^14,17^. Neonatal infections are predominantly asymptomatic and are often associated with unusual strains that show remarkable genetic stability and persist in specific settings for long periods of time^34,35^. There are geographic differences in the circulation of strains causing neonatal infections, with P[11] strains described in India and P[6] infections in Asia and Africa^35–37^. Very few studies have evaluated factors that drive the neonatal predilection of these strains or identified mediators of differences in clinical presentation between neonates. Addressing these questions at a molecular and translational level is challenging when infections occur at low frequencies or involve multiple strains of a pathogen. We focused on the G10P[11] strain that has been demonstrated to cause a high incidence of almost exclusive neonatal infections over a long period of time^15^. Having a clearly defined, age-restricted population with infection by a single strain allowed us to directly examine the role of breast milk bioactive components in infection and disease. We demonstrate previously unrecognized roles for bioactive components in breast milk in neonatal rotavirus infection and disease presentation, setting the foundation for new studies on these factors in infection with other rotavirus strains.

In order to inform clinical practice and provide guidance for the use of expressed breast milk, it is critical to understand how HMOs and/or the presence of specific bacteria translate to clinical presentations. The differences in clinical presentations are unlikely to be mediated by changes in viral load alone since the enhancement in infectivity in vitro, while significant, is modest in terms of differences in virus yield. This corresponds to data from epidemiological studies where no clear differences in viral load between symptomatic and asymptomatic neonates were observed^20^. Modulation of the microbiome and mucosal immune responses by HMOs resulting in reduction in rotavirus diarrhea with a porcine rotavirus strain has previously been demonstrated in a piglet model^38^. While the protective effects of HMOs in the piglet model contrast our findings in human neonates, it is important to note that the animal studies were carried out with a distinct and homologous porcine rotavirus strain. The host-range restriction of human-specific rotaviruses is a significant barrier for meaningful mechanistic studies in animal models. Indeed, there is currently no animal model for the human neonatal G10P[11] rotavirus strain. The profile of HMOs in fecal samples is similar to breast milk early in lactation (prior to 2 months)^1^ and is unlikely to provide mechanistic insight into the relationship between HMO utilization and microbiome composition. Delineating epithelial responses using in vitro co-culture studies to determine how specific HMOs influence rotavirus and *Enterobacter*/*Klebsiella* infectivity will be useful to understand how these factors modulate gastrointestinal disease in neonates. Technological advances in the field of physiologically relevant culture systems for enteric pathogens (such as human intestinal enteroids and organoids) are evaluating platforms that allow co-culture of pathogens with intestinal microbial communities^39^ and are likely to be valuable future tools to directly assess how the interplay between HMOs, microbiome, and rotavirus infection modulates gastrointestinal symptoms.

From preliminary mechanistic studies, it is evident that biologically relevant concentrations of HMOs can exert a direct effect on the virus (Fig. 1d). HMOs are known to resist degradation by the low pH and enzymatic environment of the stomach and reach the distal small intestine in an intact form^1^; therefore these HMO concentrations are likely to be present at the site of rotavirus infection in the human gut. Similar to an effect of lipopolysaccharide on poliovirus stability^40^, it is possible that the HMOs confer structural stability to the G10P[11] rotavirus particle and improve the efficiency of virus uptake by cells. Alternatively, it is possible that the virus potentially hijacks a cellular entry pathway utilized for HMO transport to gain entry into cells. In-depth mechanistic studies are required to dissect whether the virus acquires fitness advantage through interactions with HMOs. Our results highlight the need for choosing appropriate viral strains and HMOs when delineating human host–pathogen interactions. Fundamental questions on how specific host factors impact pathogen infectivity are often addressed using laboratory-adapted strains of pathogens. As seen in Fig. 1b, the use of a widely used simian rotavirus strain provided the opposite results (reduction in infectivity) when compared to a clinically relevant neonatal strain while there were no significant effects on globally dominant human rotaviruses belonging to the P[4] and P[8] genotypes that infect older children. By contrast, 3’-sialyllactose (3’SL) and 6’SL that showed no effect on the infectivity of the neonatal G10P[11] strain have been demonstrated to reduce the infectivity of globally dominant human rotaviruses^5^. Further, not every finding from in vitro studies translated to the population or vice versa. For example, LNnT, which also enhanced virus infectivity in MA104 cells, was not a discriminatory HMO associated with rotavirus infections in the field studies. These data underscore the importance of studying relevant strains of a pathogen and validating host factors in relevant populations when designing studies to address clinical or epidemiological questions.

There are several important implications of this work. From a broad public health perspective, the findings of this study provide new considerations for breastfeeding and childhood immunizations, particularly for rotavirus vaccines. All licensed rotavirus vaccines currently in use are live, attenuated virus strains that are orally administered early in life. While rotavirus vaccines have been introduced in nearly 100 countries, they remain less efficacious in developing countries with the highest burden of disease^41^. Two independently identified, emerging themes explaining the differences in the performance of rotavirus vaccines are the infants’ genetically determined HBGA status^42,43^ and specific differences in the intestinal microbiome^44,45^; however, these studies considered only infant factors influencing vaccine response. As can be anticipated for infants receiving vaccines at 6–14 weeks of age, maternal factors are highly likely to influence immune response to live, oral vaccines. Historically, most studies evaluating the impact of maternal factors focused only on maternal antibodies. Previous population studies evaluated breast milk immunoglobulin A, neutralizing antibodies, lactoferrin, lactadherin, and Tenascin-C in rotavirus vaccine response with varying outcomes^46–49^. The findings from our study add to an increasing body of evidence suggesting a role for other bioactive components in breast milk such as HMO and the microbiome in impacting vaccine/pathogen infectivity and immune responses^1,12,50–52^. This provides an impetus for evaluation of the role of bioactive components in breast milk in modulating rotavirus vaccine responses where a clear correlate of protection or vaccine failure remains to be identified. Indeed, increased vaccine virus replication in the presence of specific and pooled HMOs as seen with Rotavac® (Fig. 6) suggest that maternal genetics and HMO profile, with either direct effects on virus replication or indirectly through modulation of the microbiome, may be previously unrecognized factors affecting vaccine response. The relative contribution of different bioactive components may influence overall immunity as undiluted breast milk has been demonstrated to reduce P[11] infectivity in vitro^53^.

The naturally attenuated infectivity of some neonatal strains resulted in their development as new rotavirus vaccines. One neonatal strain-derived, low-cost vaccine has received WHO prequalification for global use (Rotavac) while another vaccine (RV3-BB) is currently in phase III clinical trials^33,54^. These new vaccines target developing country populations where current vaccines are less efficacious. The identification of specific HMOs or microbial communities that improve vaccine virus replication provides innovative new avenues to improve oral vaccine response and efficacy, including guiding the identification and development of specific prebiotics and probiotics that can improve the performance of oral rotavirus vaccines. While the modest increases in viral load are unlikely to impact the attenuation phenotype, even minor increases in vaccine take are likely to be highly impactful at the population level in settings of poor vaccine response. With 95% of rotavirus mortality occurring disproportionately in children in sub-Saharan Africa and Asia, improving rotavirus vaccine efficacy by even 15% could save lives of an estimated 400,000 children in the next two decades^55,56^. A parallel example has recently been described for influenza vaccines where 2’FL was found to improve innate and adaptive immune responses in a murine vaccination model^57^.

The enhancement in infectivity with individual HMOs also raises new considerations for the inclusion of specific HMOs in infant formula. Infant formula are produced predominantly from bovine milk that does not contain a similar repertoire and abundance of oligosaccharides as human milk. Clinical trials testing infant formula supplemented with 2’FL showed similar HMO uptake and growth in infants compared to breast-fed infants^58^. Large-scale preparations of 2’FL and LNnT are now available^59^. Adding to the cautionary note on the absence of data on potentially harmful effects of long-term use of a single HMO in the absence of complex mixtures present naturally in milk^60^, the findings from the present study emphasize the need for extensive evaluation of individual components before introduction for infant nutrition.

The absence of data on infant secretor status is a limitation of this study. The identification of secretor status from neonatal fecal samples is technically challenging, with the presence of maternal cells confounding the specificity of results. The collection of other relevant biological specimens such as saliva and serum from neonates was not feasible. However, infant secretor status is less likely to be a significant variable in the outcome of infection since we previously demonstrated that the VP8* domain of P[11] viruses binds developmentally regulated precursor glycans on epithelial cells whose expression is independent of infant secretor status^17^. Second, some demographic characteristics of symptomatic neonates were significantly different from the asymptomatic rotavirus-positive and rotavirus-negative neonates (Supplementary Table 1), although a previous study involving nearly 1300 neonates in the same population did not identify factors such as gestational age or mode of delivery to be associated with symptomatic rotavirus infections^15^. How these factors influence the composition of HMO and microbiome in breast milk is currently understudied. We found no significant differences in the HMO composition based on mode of delivery or gestational age of the neonate among secretor mothers. Among non-secretor mothers, while there were no significant differences in the HMO composition based on mode of delivery, the levels of 6’SL and LNT were significantly higher (analysis of variance (ANOVA), *p* < 0.05) among mothers of preterm neonates compared to term neonates. However, it should be noted that there were only 7 preterm neonates in this group limiting our ability to draw any meaningful conclusions. In addition, there were no significant differences in the abundance of *Enterobacter/Klebsiella*, *Staphylococcus*, or *Streptococcus* in breast milk based on gestational age or mode of delivery within each study group (Supplementary Figure 8). Finally, the absence of direct evidence for the role of the microbiome in symptomatic disease is another limitation. It is possible that some of the differences in infant gut microbiome are a consequence of infection and not a direct mediator of symptoms. It is also possible that a specific microbiome configuration could be required for symptomatic infection. Since it is difficult to directly determine the functional effects of the microbiome only with sequencing datasets, future studies involving co-culture of rotavirus with specific bacteria in the presence and absence of HMOs and in physiologically relevant platforms will be needed.

Overall, bridging the gap between the laboratory bench and the population, integrating molecular virology, NMR, and field studies provided new molecular and translational insight into the complex interplay between HMO, milk microbiome, and infant gut microbiome in neonatal enteric viral infection and disease. At a global level, the benefits of providing expressed breast milk far outweigh risks associated with enhanced infectivity of a unique neonatal rotavirus strain. However, the association of specific maternal factors with gastrointestinal presentations warrants further mechanistic investigation into the contribution of these factors to changes in intestinal permeability and homeostasis. Apart from addressing key questions on neonatal rotavirus infections, our findings have broad implications for neonatal enteric infections, lactation research, manufacturing infant formula, and vaccine studies.

## Methods

### Viruses, cells, and HMOs

Rotavirus infections were carried out on MA104 cells (African green monkey kidney epithelial cells, originally obtained from Dr. T. H. Flewett, Birmingham, UK)^61^. Rotaviruses are classified by a binary nomenclature system based on the genes encoding outer capsid proteins VP7 (G type) and VP4 (P type)^62^. Infectivity assays were carried out with a neonatal G10P[11] rotavirus isolate N1509 [stock titer 1.11E + 06 fluorescent focus units (FFU)/ml] that was previously adapted to cell culture by multiple passages on MA104 cells^17^. Other well-characterized rotavirus strains include simian rotavirus SA11 variant 4F SA11-4F (stock titer 8.98E + 06 FFU/ml), bovine G10P[11] rotavirus B223 (stock titer 8.22E + 05 FFU/ml), human G1P[8] rotavirus strain Wa (stock titer 9.60E + 06 FFU/ml), and human G2P[4] rotavirus strain DS1 (stock titer 5.15E + 06 FFU/ml). The MA104 cells and all rotavirus strains were serially passaged in the Estes Laboratory at Baylor College of Medicine. The G9P[11] rotavirus vaccine Rotavac® (1.0E + 05 FFU/ml) was donated for use in the study by Bharat Biotech International Ltd.

Pooled HMOs were prepared from donor milk samples using centrifugation, filtration, and chromatography as previously described^63^. Milk from >50 donors was pooled to account for heterogeneity in HMO composition between different women. HMO composition was analyzed by HPLC and mass spectrometry to ensure relative consistency of HMO composition among batches. The pooled preparation contained 22.8% LNT, 16.5% 2’FL, 10.0% LNFP I, 11.4% difucosyllacto-*N*-tetraose, 2.7% 3’SL, 1.9% LNnT, 1.6% 6’SL, 16.9% LNFP II, 1.7% sialyllacto-*N*-tetraose c (LSTc), 2.8% 3’FL, 0.4% sialyllacto-*N*-tetraose b (LSTb), 0.4% LNFP III, 1.1% disialyllactose-*N*-tetraose (DSLNT), and several other HMOs with <1% relative abundance. Pooled HMOs were lyophilized for long-term storage. Individual HMOs (2’FL, LNT, LNFP I, LNFP II, and 6’SL) were purchased from Dextra. Appropriate concentrations of lyophilized HMOs were weighed, diluted into cell culture media (0% Dulbecco’s Modified Eagle Medium), and used in infectivity assays.

### Infectivity assays

The effect of HMOs on rotavirus infectivity was assessed through standard fluorescent focus assays on MA104 cells described as previously described^5^. MA104 cells are highly permissive to rotavirus infection and are a well-established cell line for rotavirus infectivity assays in laboratories worldwide. We and others have used MA104 cells to evaluate the effects of glycans on multiple rotavirus strains, including the genotype studied in this work^5,53,61^. Briefly, confluent MA104 monolayers on 96-well plates were incubated overnight in serum-free media prior to infection. Rotavirus strains were activated with 10 µg/ml of trypsin at 37 °C for half an hour. The dilutions of virus that yielded approximately 100–200 FFU per well were suspended in media with and without HMOs at specified concentrations and allowed to bind to MA104 cells for 1 h at 37 °C. The inoculum was removed and the cells were washed once with serum-free media to remove any unbound virus. Media (with or without HMO) was then added, and the infection was allowed to continue for 15 h. Cells were then fixed with ice-cold methanol and intracellular viral antigen was detected using an anti-rotavirus polyclonal rabbit primary antibody followed by a fluorescently conjugated anti-rabbit secondary antibody (Alexa Fluor^TM^ 488-conjugated donkey anti-rabbit antibody). The number of infected cells was counted and expressed as FFU/ml. Modifications of this protocol were used to determine whether the effect of HMOs was on the virus or the cells. To determine whether HMOs had a direct effect on the virus, N1509 or SA11-4F was preincubated with pooled HMOs at 37 °C for 2 h prior to infection. Alternatively, MA104 cells were treated with HMOs overnight at 37 °C. The cells were pretreated for a longer duration when compared to pretreatment of the virus because extended incubation of Caco-2 cells in the presence of HMOs was shown to alter the expression of cell surface glycans affecting adhesion of enteropathogenic *Escherichia coli*^64^. To determine the effect of HMOs on cells, MA104 cells were washed following pretreatment with HMOs and then inoculated with virus. For effects on the virus, pretreated virus strains were inoculated on cells as described above. In both cases, HMOs were not included during the 15 h course of infection. Infectivity in the absence of HMOs served as the control in all experiments.

### Translational study design

The field component of the study was carried out in the neonatal nurseries and post-natal units of CMC, a 2700-bed tertiary care referral hospital in Vellore, India as described in previous epidemiological studies in this population^15^. Briefly, samples and clinical data were collected after informed consent was obtained from mothers of neonates hospitalized at CMC. Trained study nurses were available in the nurseries round the clock for consent, recruitment, and sample collection. Stool samples from neonates admitted in the neonatal nurseries for over 48 h with gastrointestinal symptoms (including diarrhea, vomiting, gastroesophageal reflux, gastrointestinal bleed, necrotizing enterocolitis, pneumatosis intestinalis, abdominal distension, and/or feed intolerance) were screened for rotavirus using an enzyme immunoassay (ProSpecT, Thermofisher Scientific, UK) according to the manufacturer’s instructions. Stool samples from infants admitted for >48 h in the post-natal units with non-enteric pathology or where the hospitalization was for maternal causes were tested to identify asymptomatically infected neonates and rotavirus-negative neonates. Molecular characterization of G and P types were carried out on rotavirus-positive stool samples^15^. At least one breast milk sample was collected from each mother as soon as feasible after recruitment of the mother–infant dyad. For breast milk collection, mothers were instructed to clean the breast with soap and water before collection of breast milk and then expressed breastmilk into a sterile container. We did not discard the first milk or clean with any disinfectant since babies receive the microbiome of the skin as well as that in the breastmilk. Neonates with gastrointestinal symptoms were admitted in the tertiary level nursery where pooled breast milk from mothers is given to neonates without matching of mother and neonate. Therefore, a sample of the pooled breastmilk actually given to each infant was collected for the first 10 feeds after recruitment. Since no significant differences were observed in HMO profile between different feeds (Supplementary Figure 7), data from the first sample was used for subsequent analysis. The remaining asymptomatic and rotavirus-negative neonates received direct breast milk. Demographic information on the study population is given in Supplementary Table 1.

### HMO analysis

The amount and composition of HMOs in breast milk samples was analyzed by HPLC after labeling with the fluorescent tag 2-aminobenzamide as described previously^27^. The well-established method allows for absolute quantification of the 19 most abundant and structurally distinct HMOs (that constitute >95% total HMOs), including 2’FL, 3’FL, 3’SL, LNT, LNnT, LNFP I, LNFP II, LNFP III, LSTb, LSTc, or DSLNT. Maternal secretor status was identified based on the presence or near-absence of 2’FL in breast milk.

### P[11] VP8* protein expression and NMR spectroscopy

Recombinant N-terminal glutathione *S*-transferase (GST)-tagged P[11] VP8* were expressed in *E. coli* BL21(DE3) cells (Novagen) and purified with Glutathione Sepharose 4 Fast Flow (GE Healthcare) affinity column as described previously^17,22^. The GST tag was removed by the treatment with protease thrombin overnight at 4 °C and rebinding the protein mixture to the Glutathione Sepharose column. The VP8* was further purified by size exclusion column Superdex75 (GE Healthcare) with 10 mM Tris, pH 7.4, 100 mM NaCl, 1 mM dithiothreitol at 4 °C. The concentration of the purified protein was determined by measuring absorbance at 280 nm and using an absorption coefficient of 32,430/M/cm for VP8* calculated using ProtPraram on the ExPASy server^65^. ^1^H-^15^N-labeled P[11] VP8* was purified and ligands were dissolved in 10 mM sodium phosphate, 50 mM NaCl, and 10% D_2_O buffer, pH 7. NMR experiments were performed on Avance III HD 800 MHz Ascend™ Bruker instrument equipped with quadruple resonance inverse detection QCI CryoProbe^TM^ (NMR and Drug Discovery Core, Baylor College of Medicine). 2D ^1^H-^15^N HSQC NMR experiments were collected on P[11] VP8* (70 μM) using various ligand concentrations (0.5, 2.0, 3.5, 5, 8, 20 mM). NMR spectra were analyzed using the NMRPipe^66^ and NMRFAM-Sparky^67^ software. Chemical shift changes in the ^1^H dimension for nine peaks were used to calculate *K*_d_ for the VP8:LNT glycan complex. The binding data for all glycans were globally fit to the general binding model “*ML* ↔ *M* + *L*” using the equation

$$Y = \left( {\left[ {ML} \right]/\left[ {M_{\mathrm{T}}} \right]} \right)\left( {Y_{ML}-Y_M} \right) + Y_M$$

where *M*_T_ is the total *M* concentration independent of ligation state, *Y* is Dd^1^H, *Y*_*M*_ and *Y*_*ML*_ are the binding transition baselines, and [*ML*] = (−*b* − (*b*^2^ − 4*ac*)^0.5^)/2*a*, with *a* = 1, *b* = −*K*_d_−[*M*_T_]−[*L*_T_], and *c* = [*M*_T_][*L*_T_], as described previously^68^.

### 16S rRNA gene sequencing

Bacterial genomic DNA was extracted from breast milk and stool samples using MOBIO PowerMag DNA Isolation Kit (MO BIO Laboratories; San Diego, CA). The 16S rDNA V4 region was amplified by PCR using barcoded Illumina adapter-containing primers 515F and 806R^69^ and sequenced on the MiSeq platform (Illumina; SanDiego, CA) using the 2 × 250 bp paired-end protocol yielding pair-end reads. Sequencing read pairs were demultiplexed based on the unique molecular barcodes, and reads were merged using USEARCH v7.0.1090^70^. Merging allowed zero mismatches and a minimum overlap of 50 bases, and merged reads were trimmed at the first base with a *Q* ≤ 5. In addition, a quality filter was applied to the resulting merged reads and those containing >0.05% expected errors were discarded. Sequences were stepwise clustered into operational taxonomic units (OTUs) at a similarity cutoff value of 97% using the UPARSE algorithm^71^. Chimeras were removed using USEARCH v7.0.1090. OTUs were determined by mapping the centroids to the SILVA v128 database^72^ containing only the 16S rRNA V4 region to determine taxonomies. We utilized multiple quality-control (QC) measures, including the use of non-template controls, at the microbial DNA extraction, 16S rRNA gene amplification, and amplicon sequencing processes for QC purposes. Breast milk and stool samples were processed in separate extraction and sequencing runs totaling six individual runs. Each extraction and sequencing run included a set of controls used routinely in the Alkek Center for Metagenomics and Microbiome Research (CMMR) at Baylor College of Medicine as part of its quality management program in conformity with its CLIA certification. Controls consisted of negative or blank reagent-only controls, and positive controls consisted of a previously characterized bacterial isolate carried through all the steps of the pipeline. In all cases, the positive control yielded >20,000 sequencing reads of which >99% mapped to the known bacterial isolate. All reagent-only negative controls yielded <500 sequencing reads of which <20% were mapped to bacteria. These results were in line with the guidelines established by the CMMR to maintain QC and assurance of the projects processed through the center. Resulting OTU tables were rarified to 940 reads per sample. In total, 3,047,373 reads passed QC and were successfully mapped, with 313,020 reads included in the final analysis at 940 reads/sample.

### Statistical analyses

For infectivity assays, each experimental condition was tested a minimum of three times, with technical replicates for each virus and HMO concentration within each assay. Virus titer measured in the absence of HMOs was considered to be 100% infectivity; change in virus titer in the presence of HMOs was expressed as the percentage of infectivity compared with no HMO treatment. Percentage of infectivity in the presence of each HMO was compared to the percentage of infectivity in the absence of HMOs as described previously^5^. No comparisons between different HMOs or between two concentrations of the same HMO were carried out. In the population studies, the levels of each HMO were compared between milk from mothers of symptomatic, asymptomatic, and rotavirus-negative neonates. All statistical analyses for infectivity assays and HMO profiles were performed with use of ANOVA with Dunnett’s post hoc test on GraphPad Prism version 6.0 for Windows (GraphPad Software). Multiplicity-adjusted *p* values were determined, with *p* values <0.05 considered to be statistically significant.

Microbiota data PCoA ordination plots were based on weighted UniFrac distances^73^ and significance between symptomatic rotavirus infection, asymptomatic rotavirus infection, and negative infection was determined by Permanova. Significant differences in the relative abundance of bacterial genera between groups were determined by KW test^74^. All *p* values were adjusted for multiple comparisons with the FDR algorithm and *p* values <0.05 after adjustment for multiple comparisons were considered significant. Microbiome statistics and figure generation were performed in R using the ggplot package^75^. LEfSe^30^ was performed using the Galaxy interface. LEfSe uses the non-parametric factorial KW sum-rank test to detect features with significant differential abundance between success or failure groups. LEfSe further uses LDA to estimate the effect size of each differentially abundant feature. An LDA cutoff of 4.0 and significance value (*p* value) of 0.05 were applied.

The HMO data underwent OPLS-DA using MetaboAnalyst 3.0^76^ and validated using 2000 random permutations. To determine the predictivity of individual HMOs as a marker for symptomatic rotavirus infection, ROC curves were generated in MetaboAnalyst 3.0 by linear SVM classification with Monte-Carlo cross-validation using balanced subsampling. In each Monte-Carlo cross-validation, two thirds of the samples were used to examine the feature importance and the classification model was validated using the one third of samples left out. Several iterations were performed with increasing number of HMOs to determine the optimal number of metabolites to predict symptomatic rotavirus infection, with analysis based on 2, 3, 5, 7, 10, or 19 of the top HMOs based on the selected frequency. Within- and between-subject pairwise comparisons of temporal breast milk samples was performed on samples obtained from at least four feeds based on the Bray–Curtis dissimilarity distance^77^ using the Vegan package^78^. The significance of within- and between-subject distances was calculated by Wilcox test. Statistics and figure generation was performed in R using the ggplot package^75^. MixOmics^79^ was implemented in R version 3.3 to determine the correlations between the significant HMOs and significant bacterial genera in breast milk and stool. MixOmixs uses sPLS regression and was performed in canonical mode with LASSO penalization^80^.

## Electronic supplementary material

Supplementary Information

## Data Availability

The raw sequence datasets generated and analyzed in this study are available through the Sequence Read Archive under BioProject ID: PRJNA503519. The data that support the findings of this study are available from the corresponding author upon request.
