# Supplementary material for: Human milk oligosaccharides, milk microbiome and infant gut microbiome modulate neonatal rotavirus infection
Source: Nat Commun. 2018 Nov 27;9:5010. doi: 10.1038/s41467-018-07476-4 (PMC6258677; doi:10.1038/s41467-018-07476-4)
Supplement: Supplementary file 1 — Supplementary Information [file 41467_2018_7476_MOESM1_ESM.pdf]

Supplementary Data

Human Milk Oligosaccharides, Milk Microbiome and Infant Gut Microbiome Modulate Neonatal Rotavirus Infection

Ramani et al.

Supplementary Figure 1: Enhanced infectivity of G10P[11] is not mediated by lactose that forms the reducing end of all HMOs.

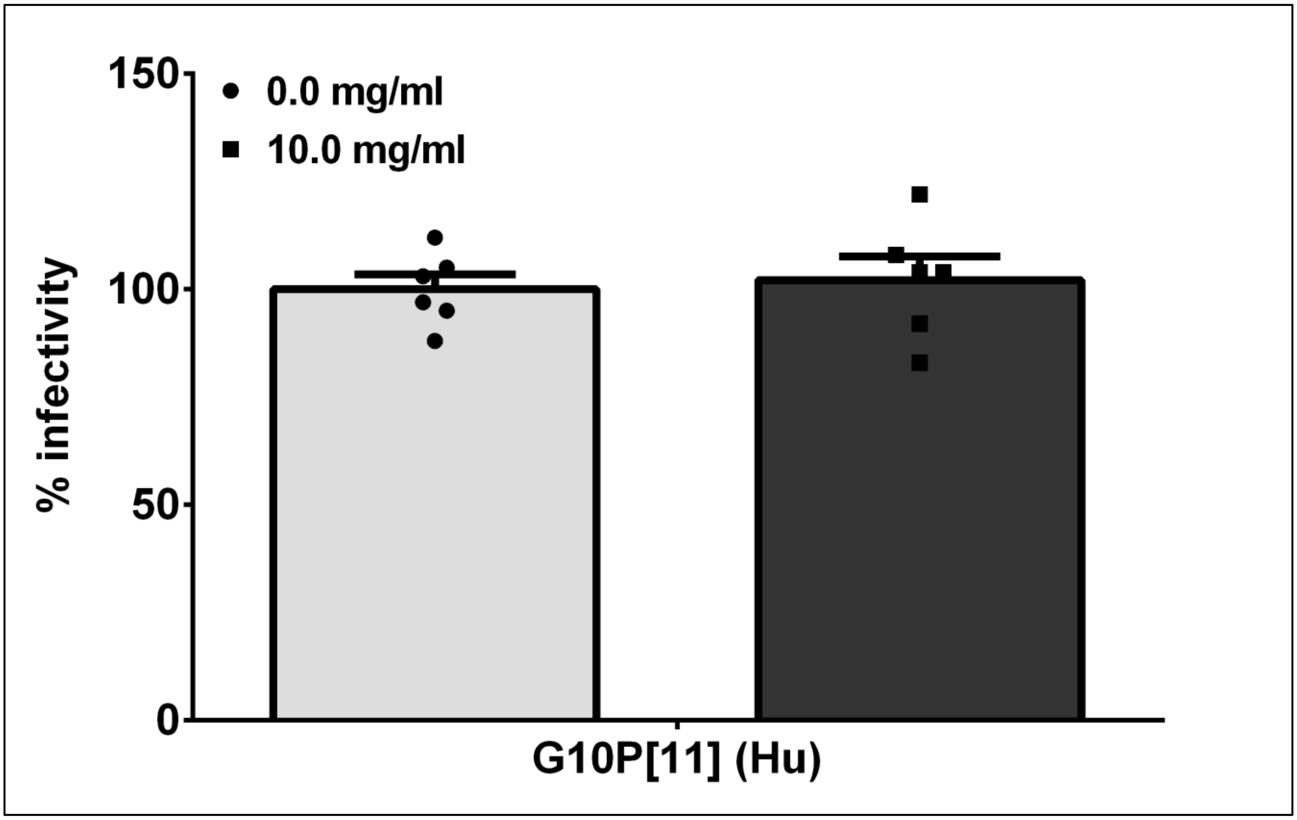

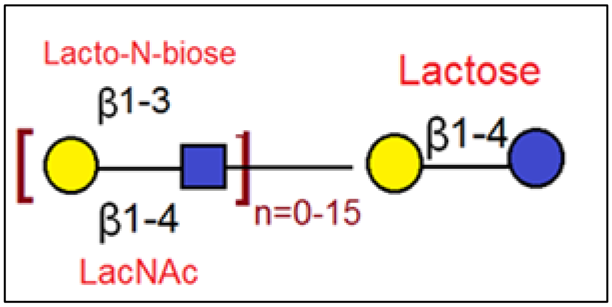

All HMOs contain lactose at the reducing end and can be extended into complex structures by the addition of type 1 (Galβ1-3GlcNAc, Lacto-N-biose) or type 2 (Galβ1-4GlcNAc, LacNAc). To determine if changes in infectivity was mediated by lactose, infectivity assays were carried out with the equivalent concentration of lactose as pooled HMOs. Bars represent the mean of % infectivity with error bars denoting standard error of the mean (n=3 experiments). No lactose treatment was considered 100% infectivity. Blue circle= glucose, yellow circle=galactose, blue square = N-acetylglucosamine

Supplementary Figure 2: Expression of HMOs varies by maternal secretor status.

a

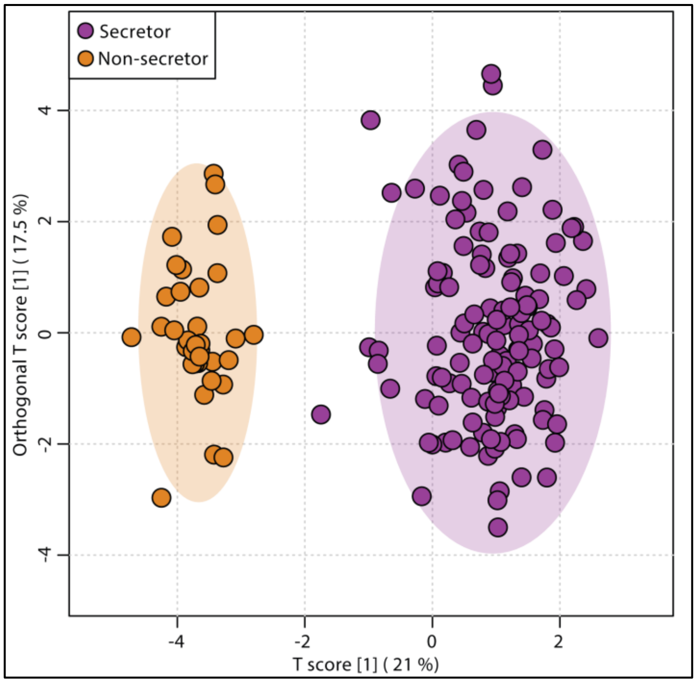

b

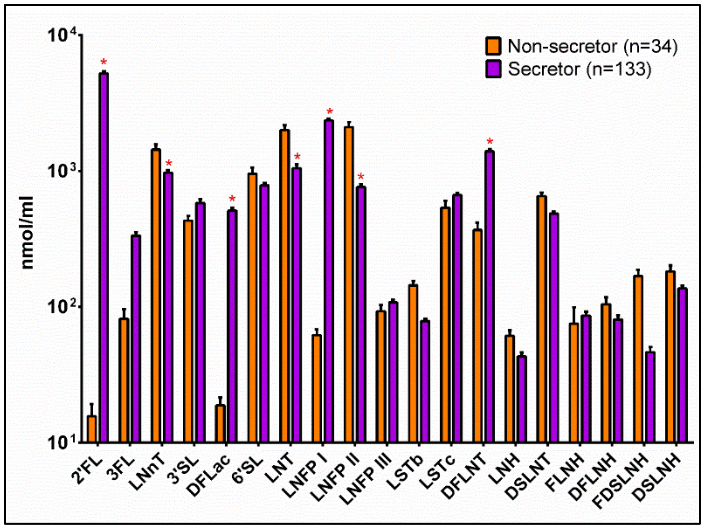

Orthogonal partial least squares-discriminatory analysis (OPLS-DA) of HMOs shows distinct differences between secretors and non-secretors (a). This distinction is mediated by significant differences in concentrations of several HMOs including 2'FL, LNnT, DFLac, LNT, LNFP1, LNFP2 and DFLNT between secretors and non-secretors (b). Bar graphs represent average levels of each HMO in nmol/ml with error bars indicating standard error of the mean. P-values <0.05 were considered statistically significant.

Supplementary Figure 3: HMO profile of breast milk is predictive of symptomatic rotavirus infections.

a

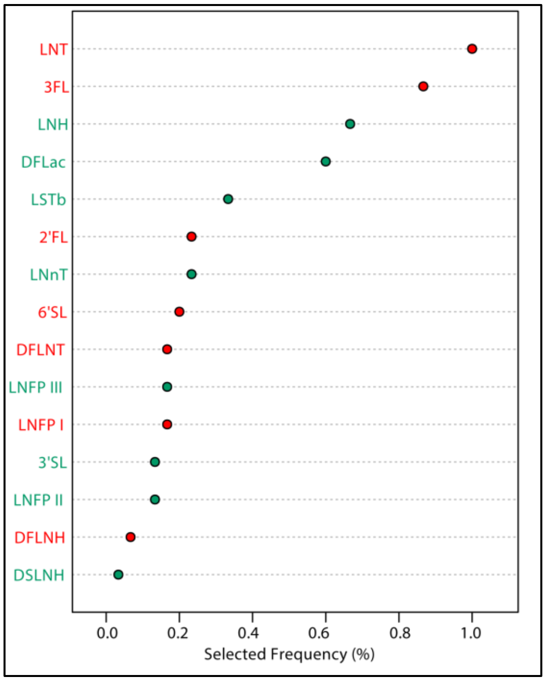

b

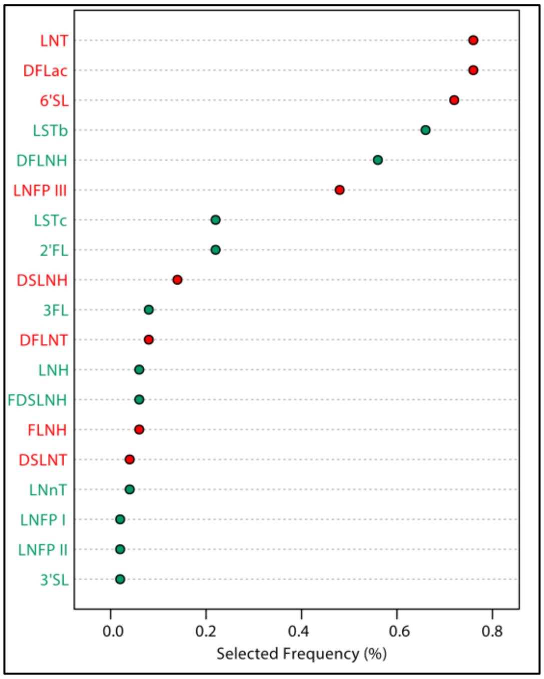

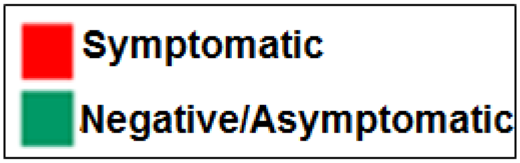

LNT was the most discriminatory HMO by linear support vector machine in both secretors (a) and non-secretors (b), with this HMO selected as one of the most important in 100% of models.

Supplementary Figure 4: NMR detects molecular interactions between P[11] VP8* and HMOs.

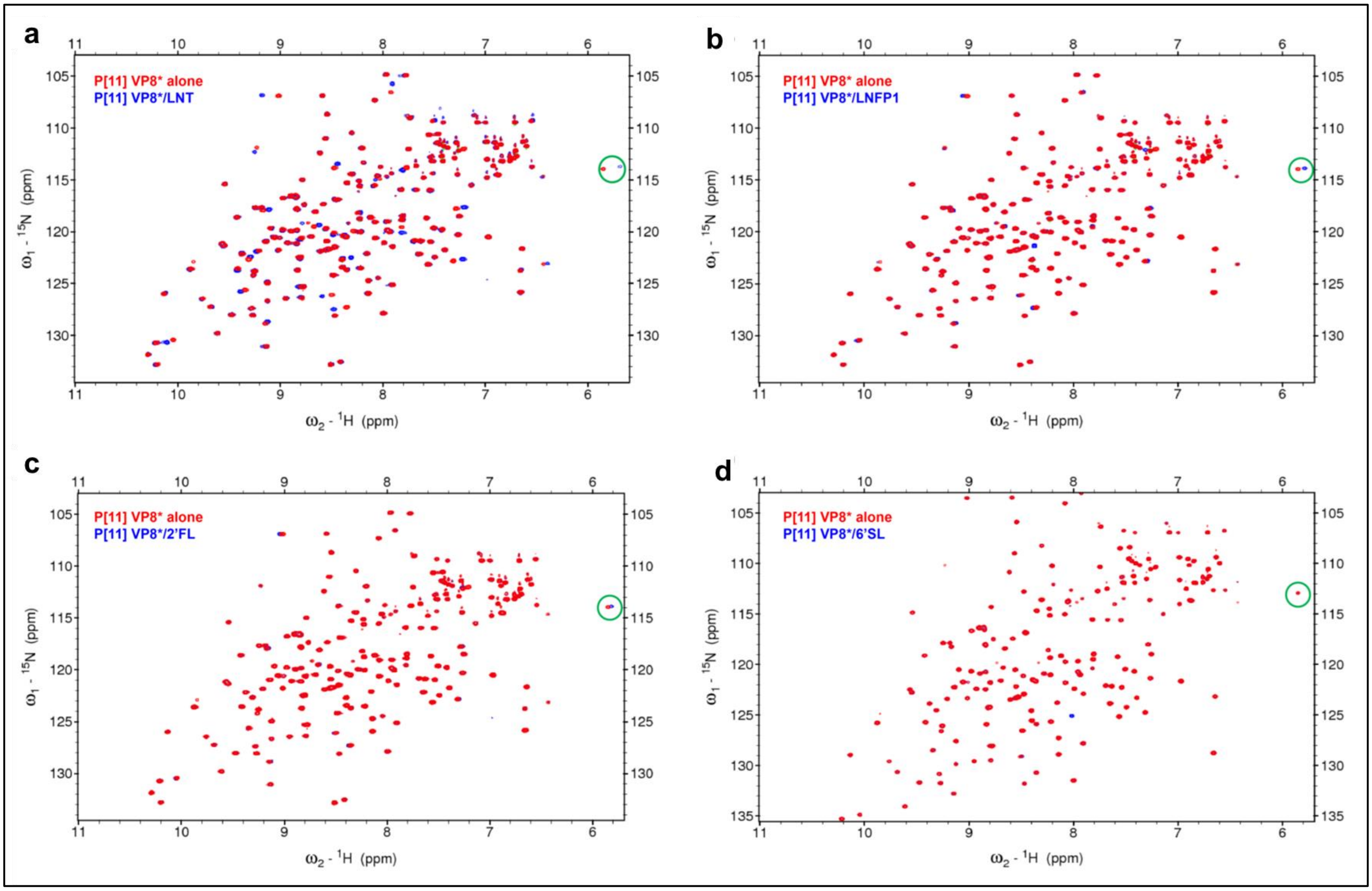

Representative 2D ^{1}H-^{15}N HSQC spectra of ^{15}N labeled P[11] VP8* alone (red) and in the presence of 20 mM (a) LNT, (b) LNFP1, (c) 2'FL and (d) 6'SL (blue) show the chemical shift changes in P[11] VP8* upon glycan binding. The largest chemical shift movements are indicated with green circles.

Supplementary Figure 5: Maternal secretor status does not influence breast milk or neonatal gut microbiome.

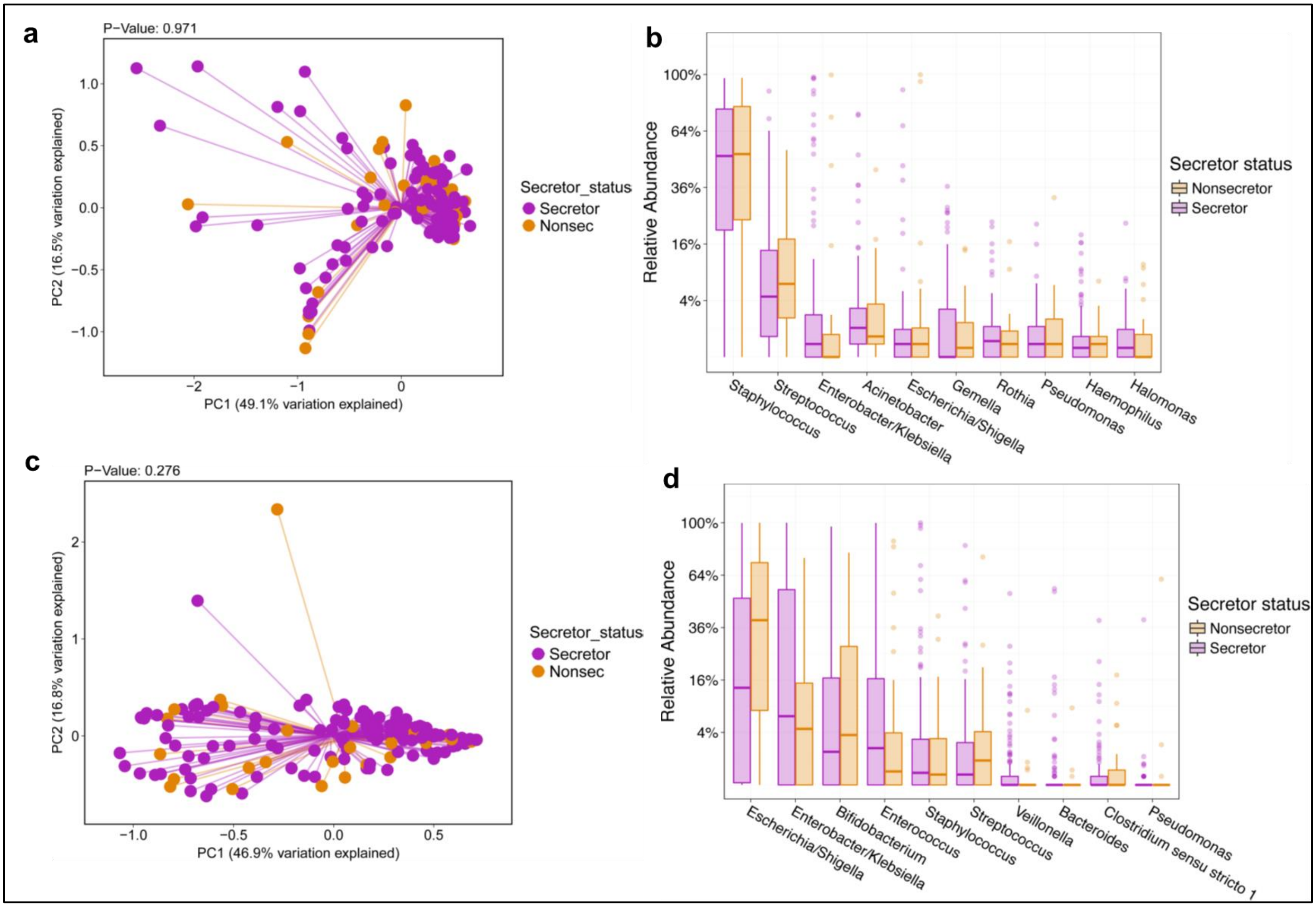

There are no differences in maternal milk (a, b) or neonatal fecal microbiome (c, d) based on maternal secretor status. The center line denotes the median, the boxes cover the 25th and 75th percentiles, and the whiskers extend to the most extreme data point, which is no more than 1.5 times the length of the box away from the box. Points outside the whiskers represent outlier samples

Supplementary Figure 6: Microbiome profile of breast milk and infant stool are also predictive of symptomatic rotavirus infections.

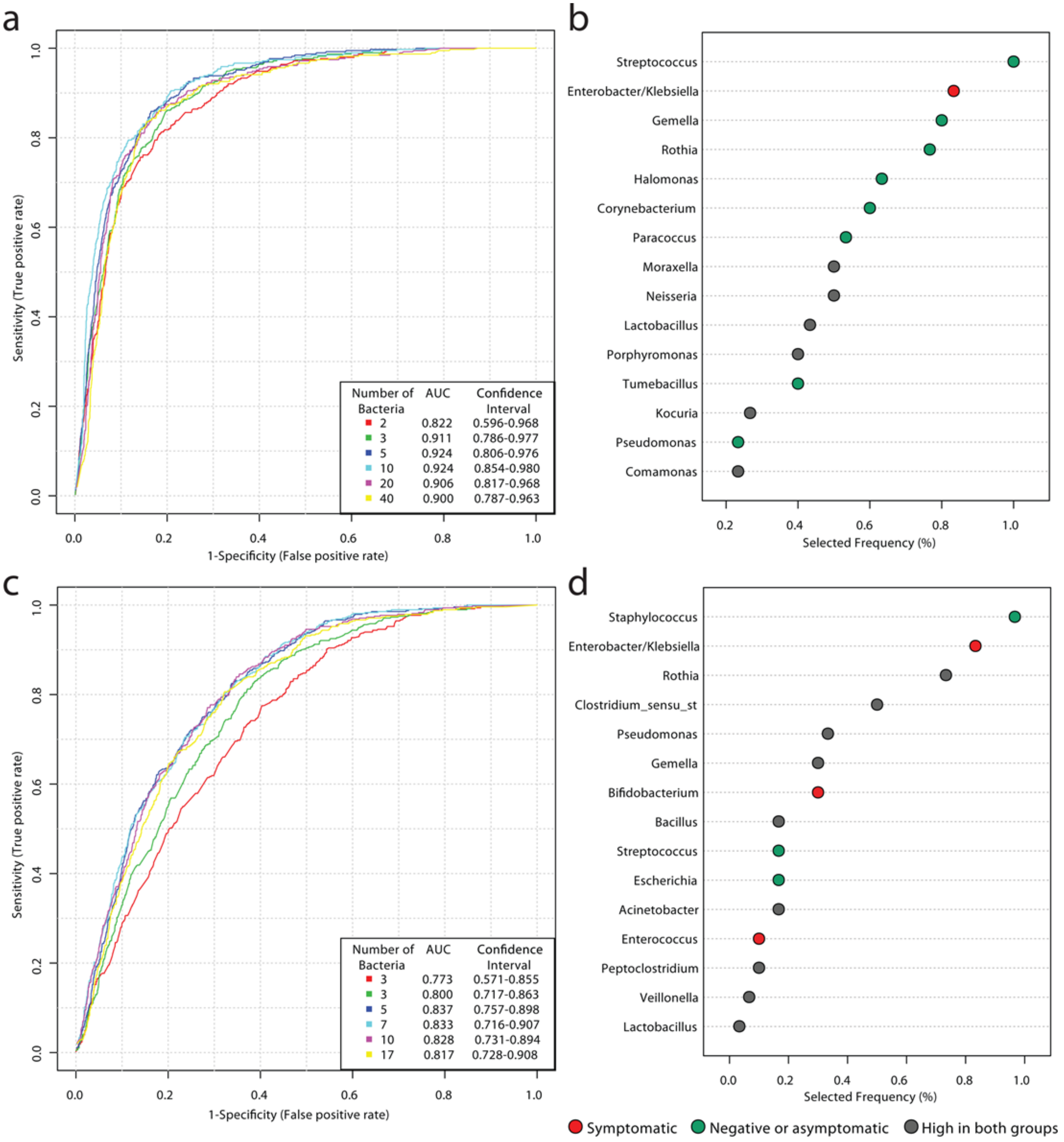

All genera over 1% in each sample type included. ROC curves generated by linear support vector machine (SVM) classification show that Enterobacter/Klebsiella was the main genera driving the prediction of symptomatic infections in breast milk (a-b) and infant stool (c-d).

Supplementary Figure 7: HMO profile of breast milk samples collected during consecutive feeds from each mother are more similar to one another (within volunteers) than to breast milk samples from other mothers (between volunteers)

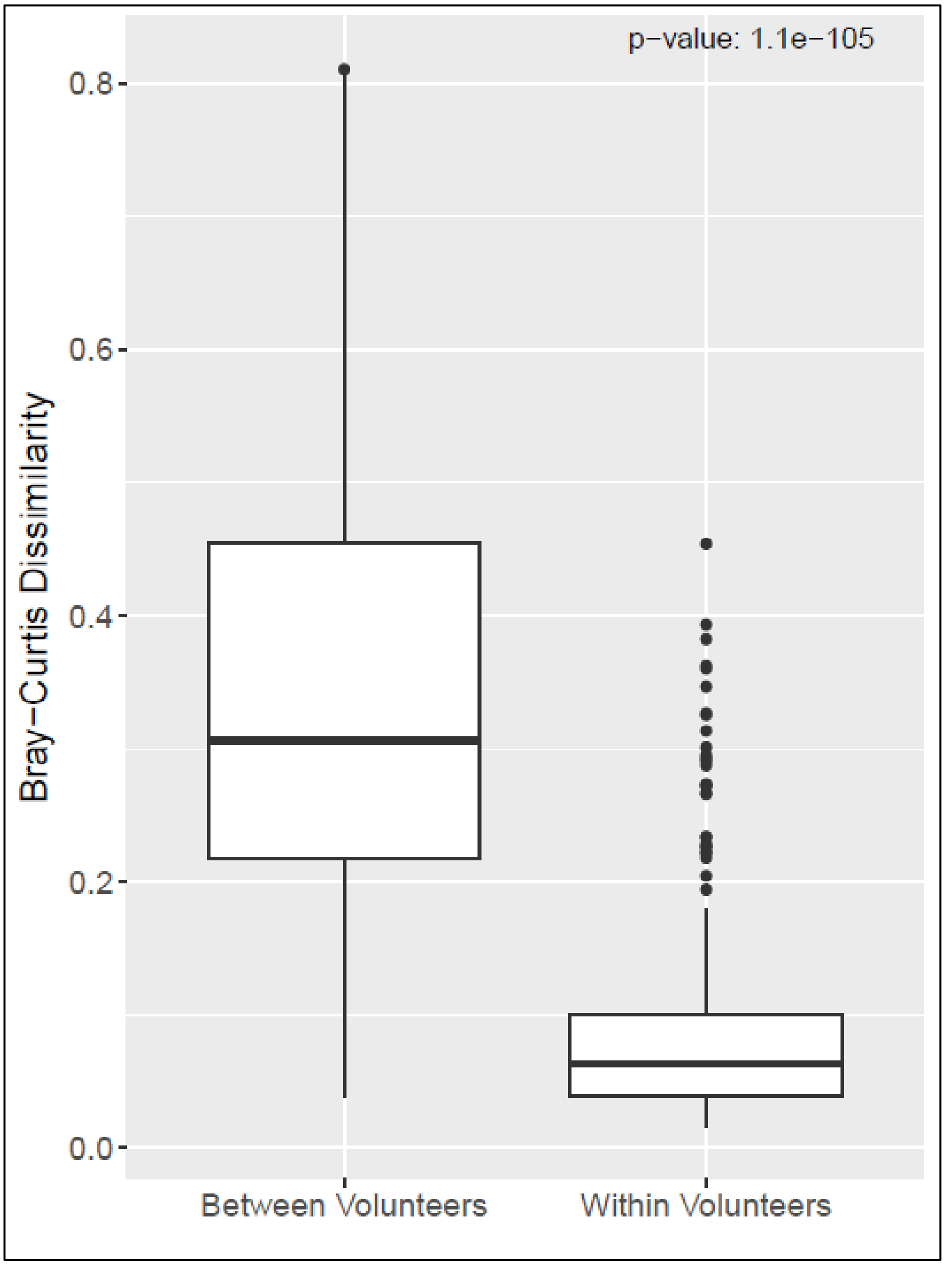

The center line denotes the median, the boxes cover the 25th and 75th percentiles, and the whiskers extend to the most extreme data point, which is no more than 1.5 times the length of the box away from the box. Points outside the whiskers represent outlier samples

Supplementary Figure 8: There were no significant differences in the abundance of *Enterobacter/Klebsiella*, *Staphylococcus* or *Streptococcus* in breast milk, based on gestational age (top panel) or mode of delivery (bottom panel) within each study group.

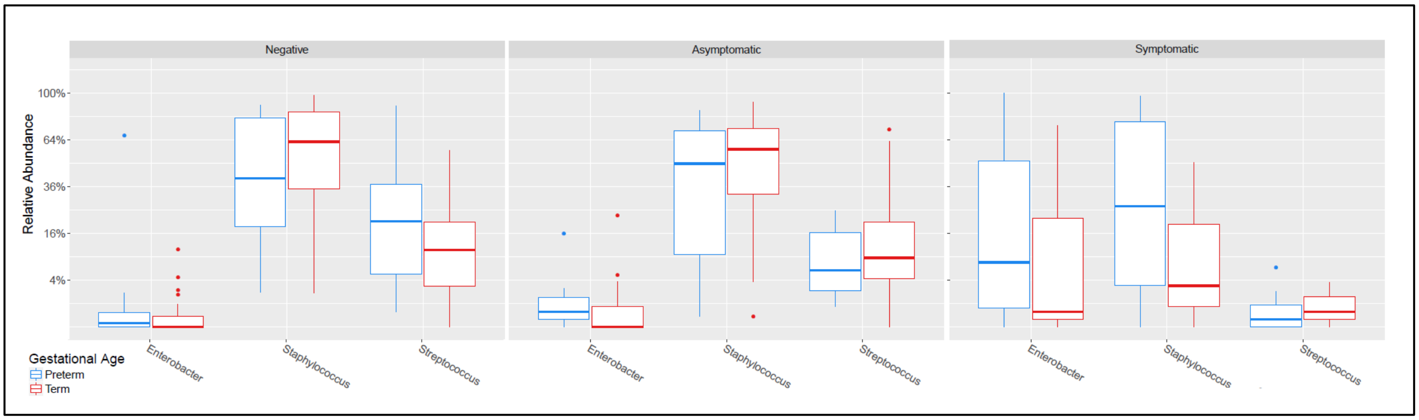

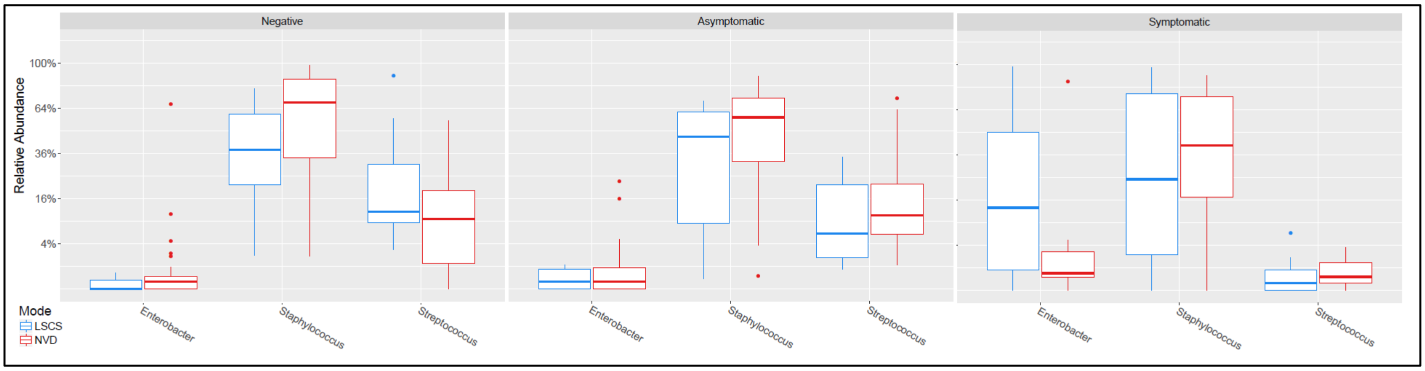

The center line denotes the median, the boxes cover the 25th and 75th percentiles, and the whiskers extend to the most extreme data point, which is no more than 1.5 times the length of the box away from the box. Points outside the whiskers represent outlier samples

Supplementary Table 1: Demographic Characteristics of Neonates Enrolled in the Study

Variable	Rotavirus negative	Rotavirus Positive		p-value*
		Asymptomatic	Symptomatic	
Total recruited (n)	65	60	56	--
HMO data available (n, % of total recruited)	63 (96.9)	55 (91.7)	49 (89.1)	0.22
Breast feeding	Direct	Direct	Expressed	--
Mean age in days (standard deviation)	3.4 (3.4)	3.8 (1.4)	11.2 (4.8)	<0.05
Female (%)	25 (38.5)	27 (45.0)	27 (48.2)	0.42
Mode of delivery (n, %)				
LSCS	15 (23.1)	14 (23.3)	35 (62.5)	
NVD	40 (61.5)	37 (61.7)	18 (32.1)	<0.05
Other	10 (15.4)	9 (15.0)	3 (5.4)	
Gestational age (n, %)				
Preterm	8 (12.3)	7 (11.7)	51 (91.1)	<0.05
Term	57 (87.7)	53 (88.3)	5 (8.9)	

* p-value comparing symptomatic rotavirus positive neonates to combined rotavirus negative and asymptomatic rotavirus positive neonates. There were no significant differences between the latter 2 groups. (LSCS = Lower segment Caesarean section, NVD = normal vaginal delivery, other = forceps, suction cup, breech delivery)
